# Device malfunction following epicardial pulsed field ablation using a focal dual energy ablation catheter in a patient with cardiac resynchronization therapy defibrillator: a case report

**DOI:** 10.1093/ehjcr/ytag811

**Published:** 2026-11-07

**Authors:** Rahin Wahedi, Julia Vogler, Stephan Willems, Arian Sultan

**Affiliations:** Department of Cardiology and Internal Intensive Care, Asklepios Klinik St. Georg, Lohmühlenstraße 5, 20099 Hamburg, Germany; Department of Cardiology and Internal Intensive Care, Asklepios Klinik St. Georg, Lohmühlenstraße 5, 20099 Hamburg, Germany; Department of Cardiology and Internal Intensive Care, Asklepios Klinik St. Georg, Lohmühlenstraße 5, 20099 Hamburg, Germany; Department of Cardiology and Internal Intensive Care, Asklepios Klinik St. Georg, Lohmühlenstraße 5, 20099 Hamburg, Germany; Faculty of Medicine, University of Cologne, Joseph-Stelzmann-Straße 20, 50931 Cologne, Germany

**Keywords:** Pulsed field ablation, Ventricular tachycardia, Epicardial ablation, Device reset, Dual-energy ablation, Device interference, Case report

## Abstract

**Background:**

Pulsed field ablation (PFA) is increasingly used, but experience with ventricular, and particularly epicardial, applications in patients with cardiac implantable electronic devices (CIEDs) remains limited.

**Case summary:**

A 49-year-old woman with LMNA- and SCN5A-associated cardiomyopathy, mildly reduced left ventricular ejection fraction (45%), and a cardiac resynchronization therapy defibrillator (CRT-D) presented with recurrent ventricular tachycardia (VT) and implantable cardioverter-defibrillator shocks. Previous endocardial VT ablation had revealed no relevant endocardial low-voltage substrate.

Repeat ablation was performed using a combined endo–epicardial approach. Epicardial mapping identified extensive low-voltage areas, with the best pace-map match at the basal lateral left ventricle. Ablation was performed using a focal dual-energy catheter with radiofrequency energy up to 40 W and PFA (2400 V).

Following epicardial PFA in proximity to the device generator and left ventricular lead, pacing abruptly changed from VVI 30/min to DDD 70/min. Initial attempts at device interrogation failed. After communication was restored, interrogation demonstrated that the CRT-D had reverted to factory settings. The device was successfully reset and reprogrammed. Further PFA near the LV lead or generator was avoided. Sensing, pacing thresholds, and lead impedances were preserved.

**Discussion:**

To our knowledge, this is the first report of a clinically relevant transient CIED malfunction temporally associated with epicardial ventricular PFA using a focal single-tip dual-energy ablation catheter. Although no permanent generator or lead dysfunction occurred, this observation highlights the potential for PFA–CIED interactions beyond conventional PFA workflows and across different catheter technologies.

Learning points

- Epicardial ventricular pulsed field ablation (PFA) using a focal dual-energy catheter may cause clinically relevant interaction with a CRT-D.
- This can include abrupt reversion from a deliberately programmed pacing mode to factory settings.
- Unexpected pacing behaviour during PFA should prompt immediate device assessment; interrogation before and after PFA is essential.
- In ventricular, and in particular epicardial PFA delivery in proximity to leads and/or generators, monitoring of device function and interrogation is crucial.

## Introduction

Pulsed field ablation (PFA) is well established for catheter ablation (CA) of atrial arrhythmias.^1,2^ With the introduction of dual catheters, toggling between PFA and radiofrequency (RF) ablation, applications are expanding beyond endocardial atrial fibrillation (AF) ablation, including endo- and epicardial ablation for ventricular arrhythmias.^3,4^ Previously, malfunction of cardiac implantable electronic devices (CIEDs) has been reported for the pentaspline catheter and for lattice-tip PFA catheters.^5^ We report a case of cardiac resynchronization therapy defibrillator (CRT-D) generator malfunction associated with epicardial PFA using a dual ablation catheter in proximity to the coronary sinus lead and the CRT-D generator.

## Summary figure

**Figure:**
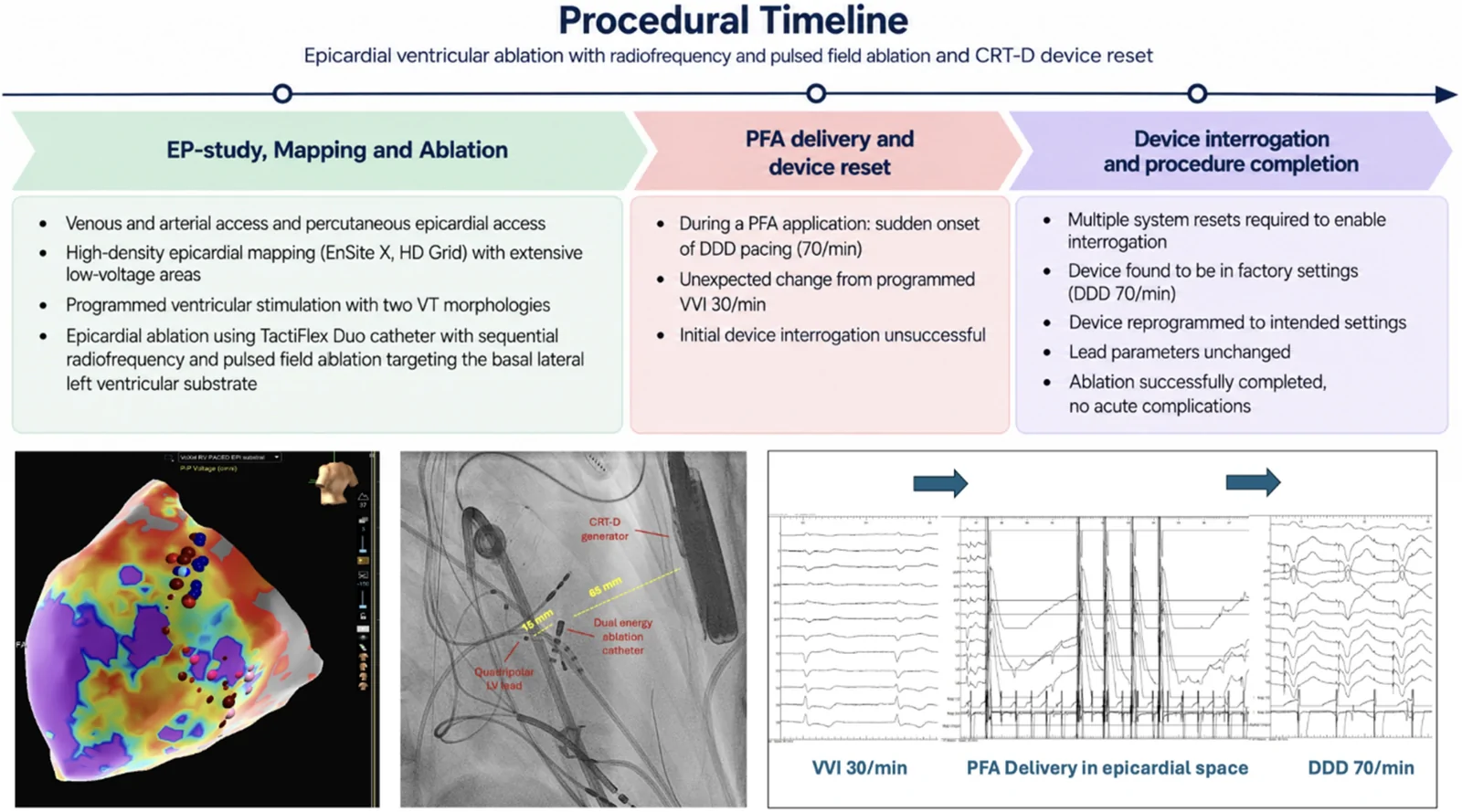


## Case presentation

A 49-year-old woman with dilated cardiomyopathy associated with pathogenic LMNA and SCN5A variants was referred to our centre for repeat CA because of recurrent ventricular tachycardia (VT) resulting in implantable cardioverter-defibrillator (ICD) shocks. A CRT-D (Rivacor 3 HF-T QP, Biotronik, Berlin) had been implanted in May 2025.

The patient had previously undergone endocardial VT ablation. During that procedure, the presumed VT substrate was localized to the lateral left ventricle in close proximity to the mitral annulus. Endocardial voltage mapping, however, did not demonstrate relevant areas of low voltage, raising suspicion of a predominantly epicardial substrate.

The patient’s medical history was additionally notable for AF and atrial tachycardia. The CHA_2_DS_2_-VA score was 1. In addition, multiple previous left atrial ablation procedures, including electrical isolation of the left atrial appendage (LAA), were carried out. This was followed by percutaneous LAA occlusion (27-mm Watchman FLX Pro, Boston Scientific, Marlborough, Massachusetts) in 2025.

After recurrence of symptomatic VT with subsequent ICD therapies and no significant substrate endocardially during the initial VT ablation, an epicardial mapping and ablation strategy was intended. Venous and arterial femoral access was obtained, followed by percutaneous epicardial access. High-density epicardial substrate mapping was performed (HDGridX mapping catheter, Abbott, Abbott Park, IL, USA) in conjunction with electroanatomical mapping (EnSiteX, Abbott, Abbott Park, IL, USA). In contrast to the findings during the previous endocardial procedure, epicardial voltage mapping demonstrated extensive areas of low voltage (*Figure 1*) with distinct late potentials. Pace mapping identified the best morphological match to the clinical VT at the basal lateral left ventricle, consistent with an epicardial arrhythmogenic substrate.

**Figure 1:**
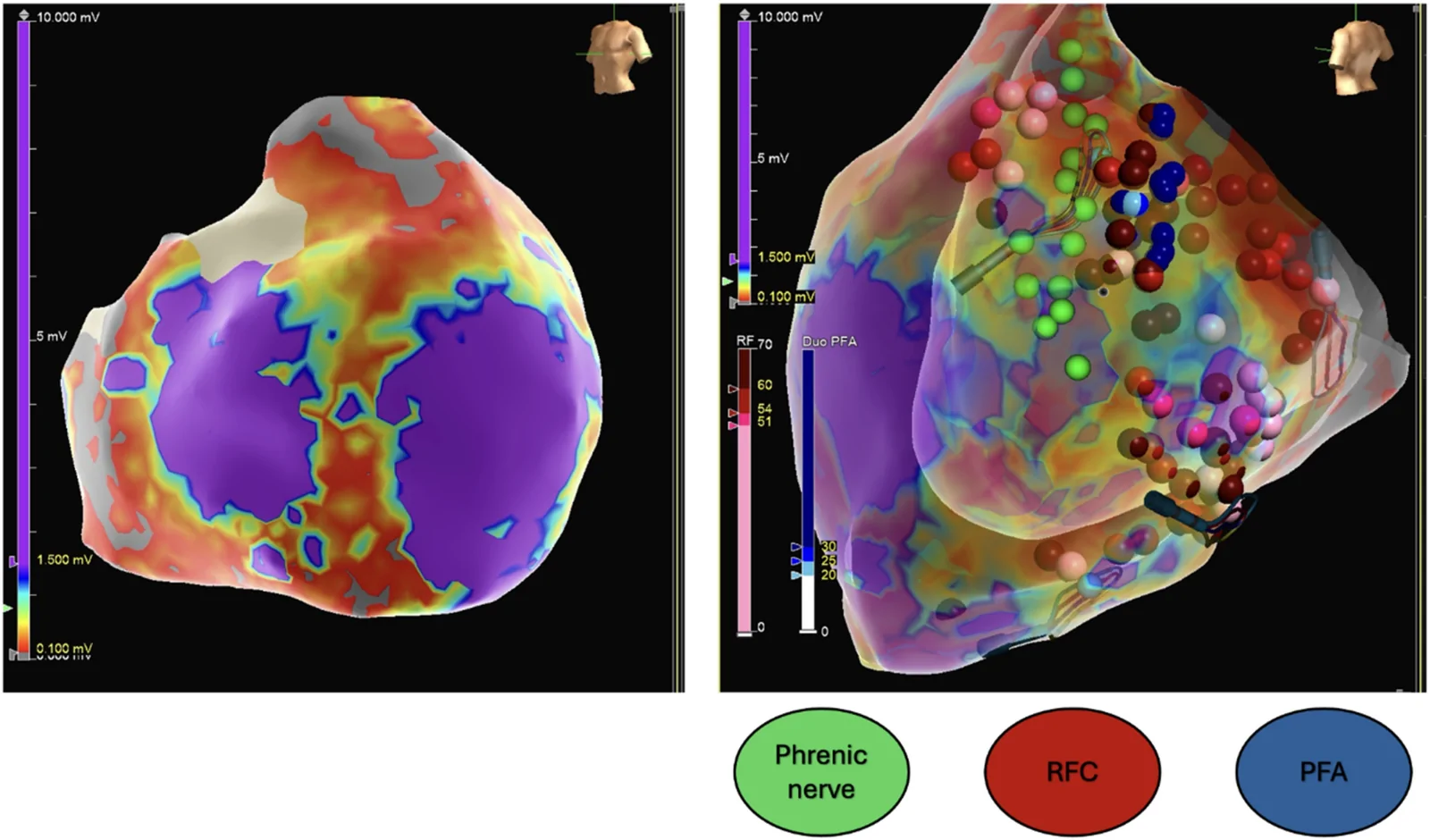
Epicardial substrate and ablation map. Epicardial voltage mapping demonstrated extensive abnormal low-voltage substrate. The corresponding ablation map shows the distribution of radiofrequency and pulsed field applications, with the best pace-map region localized to the basal lateral left ventricle.

Epicardial ablation was subsequently performed using a dual-energy ablation catheter (TactiFlex Duo catheter, Abbott; Abbott Laboratories) with both RF energy up to 40 W and repetitive PFA (monopolar biphasic waveform, 2400 V, *Figure 2*). Following PFA application in close vicinity to the LV lead and/or the laterally implanted CRT-D generator, sudden onset of DDD pacing was observed (immediately after the PFA train, DDD pacing started, *Figure 3*). Attempts to interrogate the CRT-D were initially unsuccessful despite multiple attempts. Several resets of the device interrogation system were required before communication with the CRT-D could be re-established permanently.

**Figure 2:**
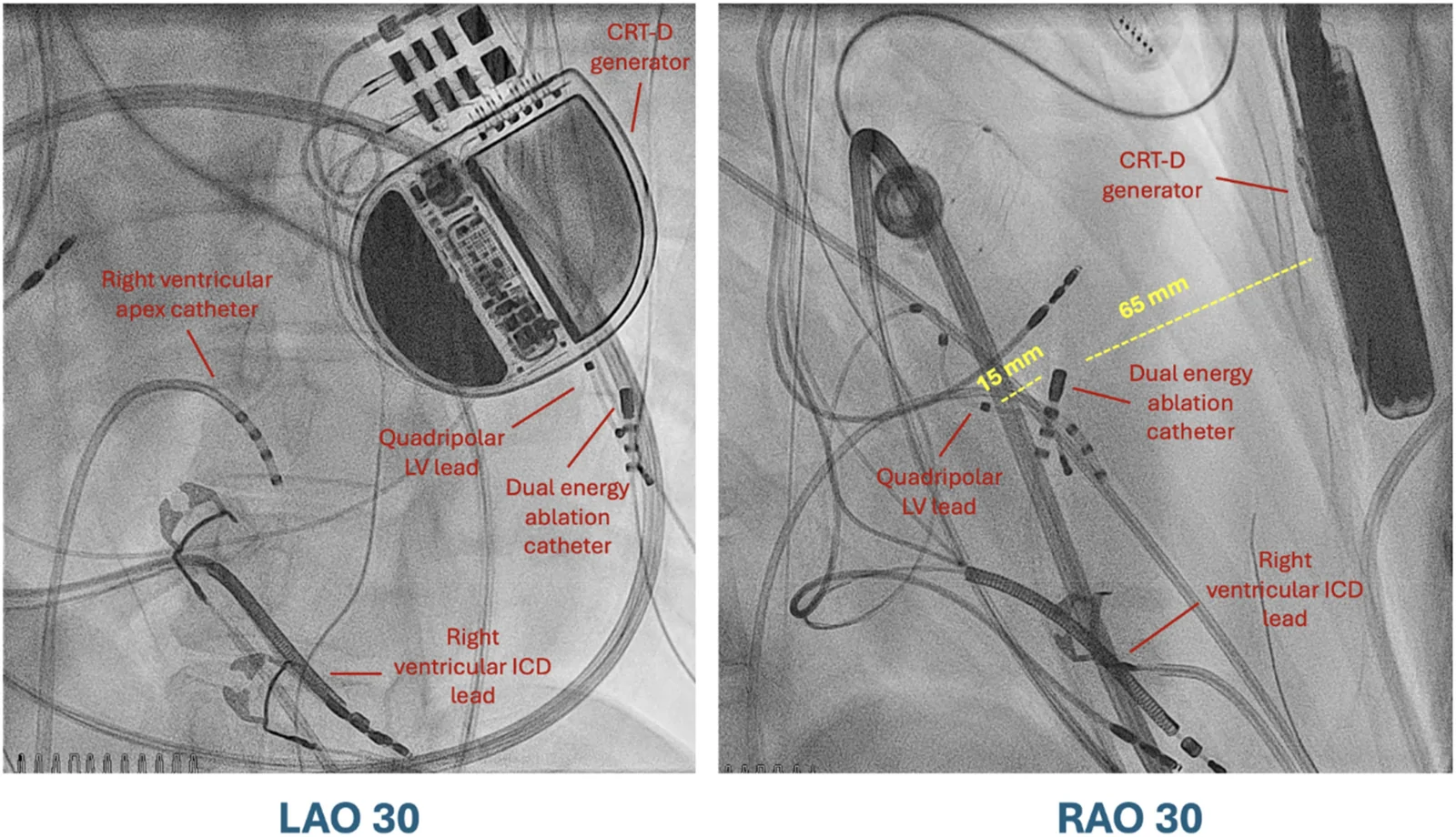
Fluoroscopy showing the spatial relation of the focal dual ablation catheter and the coronary sinus-lead as well as the cardiac resynchronization therapy defibrillator generator. Distance between the focal dual ablation catheter and the coronary sinus-lead/cardiac resynchronization therapy defibrillator generator in left anterior oblique (LAO) and right anterior oblique (RAO) views during the pulsed field ablation delivery leading to the cardiac resynchronization therapy defibrillatormalfunction.

**Figure 3:**
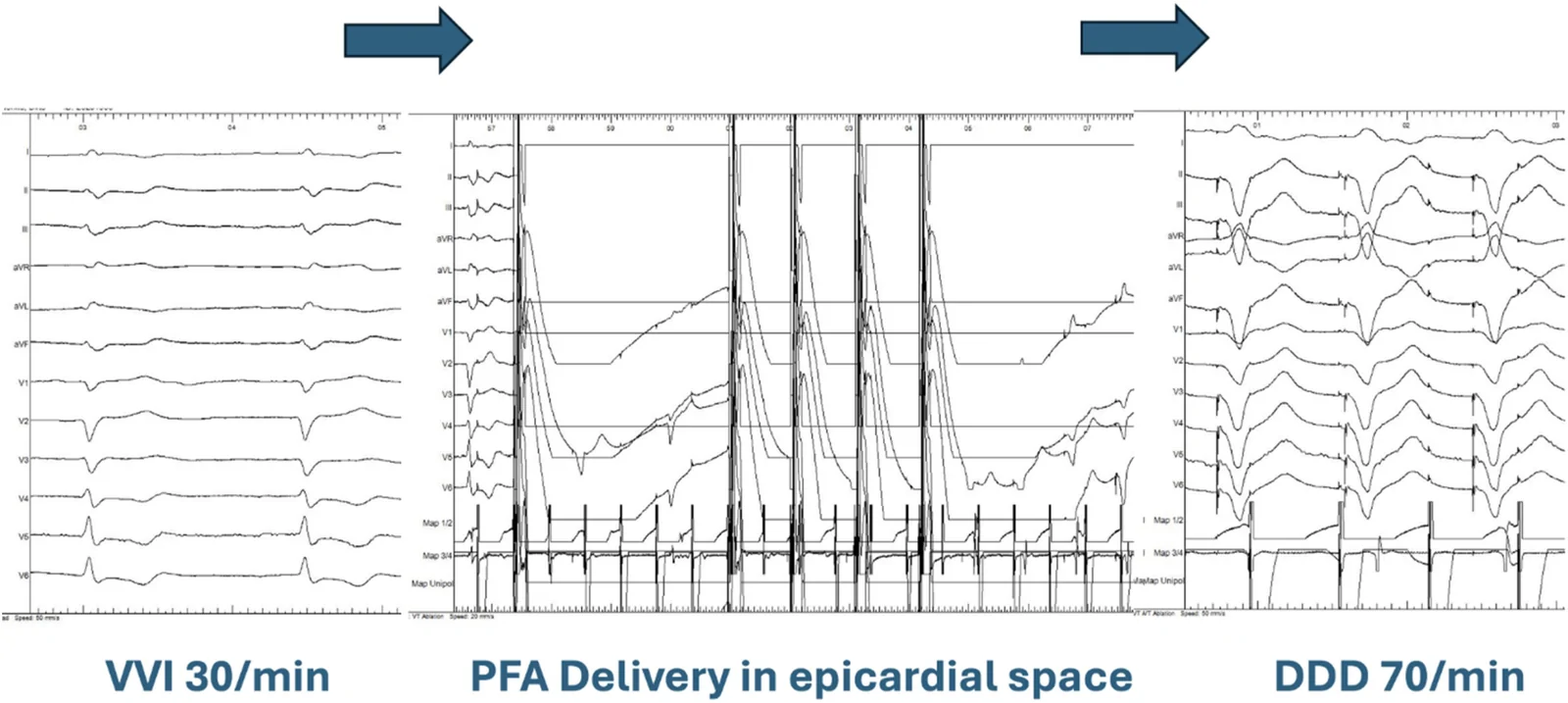
Pulsed field ablation and subsequent change in cardiac resynchronization therapy defibrillator pacing behaviour. Electrical recording during epicardial pulsed field ablation delivery. Subsequent tracing demonstrating DDD pacing at 70 b.p.m. The cardiac resynchronization therapy defibrillator had been intentionally programmed to VVI 30 b.p.m. before ablation; later interrogation demonstrated reversion to factory settings.

After successful interrogation, the CRT-D was found to have reverted to factory settings. The device parameters could subsequently be reset and reprogrammed to the intended settings. After restoration of device communication and programming, the ablation procedure was continued and successfully completed but omitting PFA applications in close vicinity to LV lead or generator.

The final lead parameters, including sensing, pacing thresholds, and impedances, were unchanged from baseline. No further device malfunction occurred during the procedure.

## Discussion

Pulsed field ablation has emerged as an alternative to conventional ablation by inducing irreversible electroporation through the delivery of high-voltage electrical pulses.^6–8^ While its clinical use has predominantly focused on endocardial ablation for AF, potential applications of PFA are expanding to more complex arrhythmogenic substrates, including ventricular and epicardial ablation.^9^ With this expansion, interactions between PFA and CIEDs represent an increasingly relevant safety consideration. We report a case of transient CRT-D malfunction with reversion to factory settings occurring following epicardial PFA.

Available clinical experience in AF ablation suggests that PFA can generally be performed safely in patients with pacemakers and implantable cardioverter-defibrillators.^10^ Nevertheless, the high-voltage electrical fields generated during PFA have the potential to interact with CIEDs. Previously described effects include transient electromagnetic interference, oversensing, inhibition of pacing, and inappropriate detection of electrical signals.^5^ Importantly, most available experience has been derived from endocardial PFA, particularly pulmonary vein isolation, where the ablation site is generally spatially separated from ventricular pacing and defibrillation leads.

The present case differs from conventional PFA procedures and already reported cases of device malfunction after PFA/RF application in close proximity to a CIED in several important aspects. First, to our knowledge, this is the first reported temporal malfunction of this kind after the use of a focal dual-energy ablation catheter. Second, PFA was delivered epicardially at the basal lateral left ventricle, potentially resulting in a different spatial relationship between the electrical field and components of the CRT-D system. Third, due to the CRT-D, the left ventricular pacing lead is positioned ‘epicardially’ and therefore potentially more prone to interactions. Therefore, depending on the position of the LV lead and other components of the device system, PFA may have temporally exposed the CIED to a substantial electrical field. Finally, the observed event occurred immediately after PFA delivery, supporting a temporal association between energy application and device malfunction.

Notably, the device could initially not be interrogated despite repeated attempts. After communication was restored following several system resets, interrogation demonstrated reversion of the CRT-D to factory settings, as suggested by the DDD pacing mode rather than VVI as seen in backup safety modes.^11^ The device could subsequently be reprogrammed, and post-procedural interrogation demonstrated normal function without persistent abnormalities of sensing, pacing thresholds, or lead impedances. These findings suggest a transient interaction affecting the device rather than structural damage to the generator or leads. However, the precise mechanism responsible for the reset cannot be established from this single case. A direct effect of the PFA electrical field on the pulse generator, electromagnetic interference involving the leads, or activation of an internal device safety mechanism should be considered as potential explanations. It may also be the case that further applications may have caused irreversible damage to leads and/or the generator.

In patients with CIEDs undergoing endo- and/or epicardial PFA, device integrity should be monitored during the procedure foremost after applying energy in close proximity to any CIED components. In addition, the location of the CIED in relation to the intended ablation site should be considered during procedural planning. Whether specific minimum distances between PFA applications and CIED leads or other modifications of energy delivery are required remains unknown and should be addressed in dedicated experimental and clinical studies.

This case highlights a potentially important interaction between epicardial PFA and a CRT-D, characterized by transient loss of device communication and reversion to factory settings immediately following PFA delivery. Although normal device function could be restored without evidence of permanent damage, the event emphasizes the need for careful peri-procedural device surveillance when PFA is used for ventricular and particularly epicardial VT ablation. Further investigation is required to determine the mechanisms, incidence, and appropriate preventive strategies for PFA-related interactions with CIEDs.

## Conclusion

This case demonstrates a potentially important interaction between epicardial PFA using a focal dual-energy ablation catheter and a CIED. As PFA applications expand beyond endocardial pulmonary vein isolation, systematic evaluation of device safety, particularly during epicardial energy delivery, is warranted.

## Lead author biography


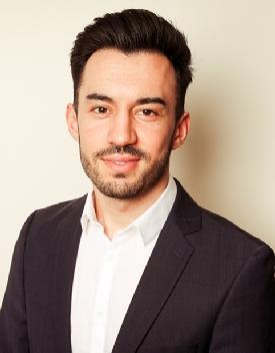


Rahin Wahedi, MD, is a physician and clinical researcher at the Department of Cardiology and Internal Intensive Care at Asklepios Klinik St. Georg in Hamburg, Germany. His clinical and scientific interests focus on cardiac electrophysiology, CA, and the management of atrial and ventricular arrhythmias. His research includes novel ablation technologies, particularly PFA, as well as outcomes of CA in patients with CIEDs. His current work focuses on the clinical implementation and safety of emerging ablation technologies and their application in increasingly complex arrhythmia substrates.

## Data Availability

All relevant data are within the manuscript. No data have been shared.
